# Tissue Multicolor STED Nanoscopy of Presynaptic Proteins in the Calyx of Held

**DOI:** 10.1371/journal.pone.0062893

**Published:** 2013-04-26

**Authors:** Christian Kempf, Thorsten Staudt, Pit Bingen, Heinz Horstmann, Johann Engelhardt, Stefan W. Hell, Thomas Kuner

**Affiliations:** 1 Institute of Anatomy and Cell Biology, Heidelberg University, Heidelberg, Germany; 2 Department of Nanobiophotonics, Max Planck Institute for Biophysical Chemistry, Göttingen, Germany; 3 Bioquant Center, University of Heidelberg, Heidelberg, Germany; 4 Optical Nanoscopy Division, German Cancer Research Center (DKFZ), Heidelberg, Germany; CNRS - Université Aix Marseille, France

## Abstract

The calyx of Held, a large glutamatergic terminal in the mammalian auditory brainstem has been extensively employed to study presynaptic structure and function in the central nervous system. Nevertheless, the nanoarchitecture of presynaptic proteins and subcellular components in the calyx terminal and its relation to functional properties of synaptic transmission is only poorly understood. Here, we use stimulated emission depletion (STED) nanoscopy of calyces in thin sections of aldehyde-fixed rat brain tissue to visualize immuno-labeled synaptic proteins including VGluT1, synaptophysin, Rab3A and synapsin with a lateral resolution of approximately 40 nm. Excitation multiplexing of suitable fluorescent dyes deciphered the spatial arrangement of the presynaptic phospho-protein synapsin relative to synaptic vesicles labeled with anti-VGluT1. Both predominantly occupied the same focal volume, yet may exist in exclusive domains containing either VGluT1 or synapsin immunoreactivity. While the latter have been observed with diffraction-limited fluorescence microscopy, STED microscopy for the first time revealed VGluT1-positive domains lacking synapsins. This observation supports the hypothesis that molecularly and structurally distinct synaptic vesicle pools operate in presynaptic nerve terminals.

## Introduction

Understanding the fundamental principles that govern neural signaling is one of the foremost challenges in neuroscience. Given the complexity and velocity of molecular interactions mediating neurotransmitter release, these processes must be coupled to a precise functional geometry on the nanometer scale within active zones and their corresponding synaptic vesicle clusters [1], [2]. While the molecular composition of presynaptic nerve terminals has been elucidated at quite some detail [3], the exact distribution and geometrical arrangement of proteins on the scale of nanometers and the impact of this organization on synaptic function remain mostly unknown. This lack of progress is mostly due to the fact that conventional fluorescence microscopy is limited to a spatial resolution of 250 nm×250 nm in the focal plane (x-y) and >600 nm along the optical axis (z). Hence, it is not suitable for the precise imaging of protein topology (few nm), synaptic vesicles (50 nm) or active zones (500 nm). These limitations have been overcome by fluorescence super-resolution imaging techniques, which maintain key advantages of fluorescence microscopy such as the simultaneous detection of multiple labels. The advance from single cells or monolayer cell cultures to complex brain tissue imaging represents a significant challenge. In this work, we present the first application of single- and multicolor STED microscopy [4] on synaptic organization in aldehyde-fixed mammalian brain tissue.

**Figure 1:**
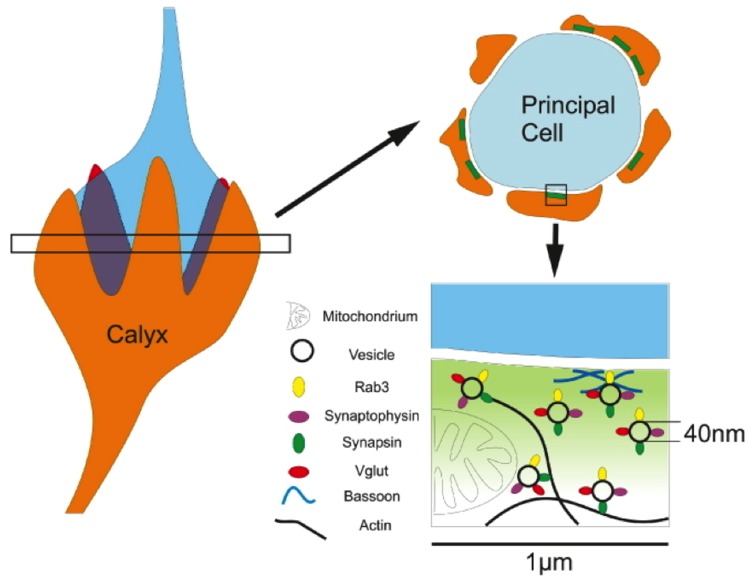
Schematic representation of the calyx of Held’s architecture. Diagram illustrating the location of a typical 4 µm brain slice (open bar) with regard to the 3D structure of the calyx of Held (orange) and its postsynaptic cell (blue). A typical cross section through the principal cell and calyx reveals a ring-like arrangement of calyx segments along the circumference of the principal cell (upper right). Active zones (green) are located along the release face of the calyx segments adjacent to the principal cell. The hypothesized localizations of the synaptic proteins investigated in this study are depicted in relation to mitochondria, the presynaptic cytoskeleton and the presynaptic membrane. This ring-like arrangement of presynaptic components illustrates the expected distribution of immunostainings shown in Figures 2 to 7.

To investigate the applicability of STED microscopy in neuronal tissue samples, we selected the calyx of Held, a giant terminal in the medial nucleus of the trapezoid body located in the auditory brain stem (**Figure 1**). This glutamatergic terminal (orange) wraps around the large spherical principal cell measuring 20 µm in diameter (blue) and forms a large contact area with a membrane surface of approximately 1000 µm^2^, which harbours several hundred active zones (green) [5], [6]. It offers the challenge to visualize a large structure yet at the same time resolve compartmentalized geometrical units on the nanoscale (Figure 1, lower right). Furthermore, the calyx offers unique access to the biophysics of synaptic transmission [7], hence providing a model system to relate nanoscopic architecture to synaptic function.

Using immunohistochemistry we show the distribution of several synaptic vesicle proteins including the vesicular glutamate transporter (VGluT1), synaptophysin, Rab3A and synapsin. We furthermore used dual-color STED microscopy to visualize the distribution of synapsin relative to immuno-labelled synaptic vesicles, probing the question if synapsin proteins delineate a subset of the reserve pool of synaptic vesicles.

## Results

### Tissue STED Nanoscopy Reveals Immuno-labeled Synaptic Vesicles at 40 nm Focal Plane Resolution

**Figure 2:**
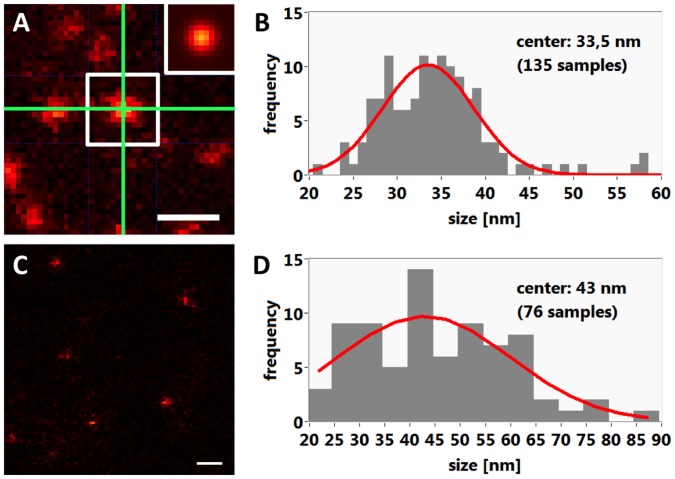
Determination of the STED microscope’s resolution. (**A**) Image of Nilered beads adsorbed to a glass surface. The center of the green crosshair marks the central point from which twodimensional gauss functions were fit. The inset shows the fitted gaussian distribution of the fluorescence intensities. Scale bar 100 nm, pixel size 8.57 nm. (**B**) Histogram of the size distributions measured from Nilered beads as described in A. The mean bead size was 33.5 nm, consistent with a diameter of 33 nm theoretically predicted (63×1.3 NA oil, excitation 532 nm, emission 580 nm, STED 647 nm delivers FWHM 30 nm at a 120 mW (80 MHz) STED beam; beads with a physical diameter of 25 nm will then appear to be 33 nm wide). (**C**) Mowiol-embedded IgG antibodies conjugated to Atto565 adsorbed to a glass surface. 2D-fits were only applied to non-overlapping spots. Scalebar 200 nm. (**D**) Histogram of the size distributions measured from antibodies using the method described in A. The mean spot size was 43 nm.

We first determined the resolution of the microscope by imaging Nilered beads with a diameter of 25 nm. Using a 2D gaussian fit procedure (Fig. 2A) we determined a resolution of 33.5 nm (Fig. 2B), close to the theoretically expected value of 33 nm (see legend of Figure 2). Each bead hosts many dye molecules giving rise to a good signal to noise ratio with a small apparent measured size variation and almost no blinking effects. This does not apply for antibodies which carry only a few dye molecules, causing a low signal to noise ratio and significant blinking. To obtain the instrument resolution for antibody-mediated localization we imaged a sheep anti-mouse IgG antibody labeled with Atto565 adsorbed to a glass surface. After embedding in Mowiol, single point-like objects could be readily identified and revealed a full-width-half-maximum (FWHM) resolution of 43 nm (**Figure 2C,D**).

**Figure 3:**
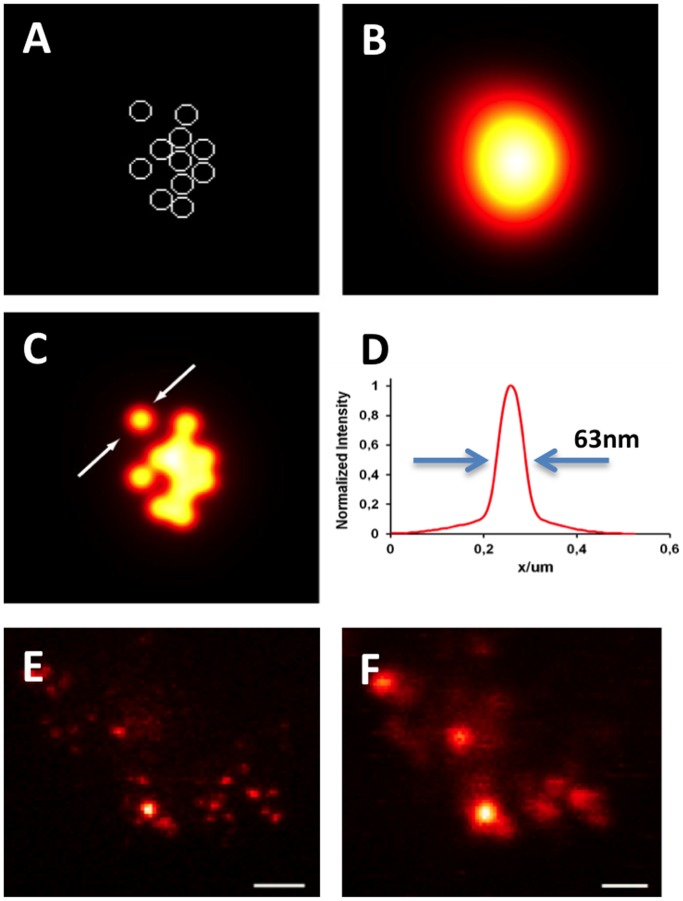
Simulation and imaging of synaptic vesicle clusters in the calyx of Held. (**A**) Arbitrary cluster of synaptic vescles with a diameter of 50 nm. (**B**) Illustration of the theoretically expected confocal (**B**) and STED (**C**) images of the synaptic vesicle cluster with an assumed resolution of 200 nm and 43 nm, respectively. (**D**) Profile across the arrows indicated in C reveals FWHM of 63 nm. (**E**) Raw STED image frame showing individual synaptic vesicles labeled with an anti-VGluT-antibody in a 4 µm thick PFA-fixed brain slice. The average size measured with 2D gauss fits was 61 nm. (**F**) Confocal image corresponding to E. Scale bars 500 nm.

Next, the expected STED microscopy image of an arbitrary vesicle distribution was mathematically simulated by assuming the synaptic vesicles (including protein and label) to be hollow spheres with a diameter of 50 nm (**Figure 3A**) convolved with a confocal PSF of ∼200 nm for confocal measurements (**Figure 3B**) and a 43 nm (FWHM) Gaussian PSF for STED microscopy (**Figure 3C**). The predicted size of a 50 nm vesicle imaged with STED microscopy is 63 nm (**Figure 3D**). This analysis suggests that single vesicles can be distinguished using STED microscopy even in areas with high vesicle densities that can not be resolved with confocal microscopy.

This simulation was compared to STED microscopy in the physicochemical environment of mammalian neuronal tissue, for which we extracted paraformaldehyde-fixed brainstems of transcardially-perfused rats at the age of P14–16. STED and confocal microscopy images of the anti-VGluT1 labeled tissue sample containing the calyx of Held giant presynaptic terminal were acquired (**Figure 3E,F**). STED microscopy revealed solitary signals with a FWHM of ∼61 nm in the focal plane, consistent with the size of single vesicles simulated with the 43 nm resolution obtained from measurements done on glass-adsorbed antibodies shown above, yielding a size (FWHM) of 61±10 nm (mean±SD). When using Dyomics485×l-labelled antibodies, we obtained an average vesicular diameter of 94±20 nm (mean±SD). This was expected because of the reduced signal-to-noise conditions encountered with this dye compared to Atto565 (not shown).

Thus, we successfully demonstrated the potential of STED microscopy to resolve fluorescence signals in mammalian brain tissue with a focal plane resolution around the size of a single synaptic vesicle (**Figure 3**). Because the axial resolution remains at around 600 nm, synaptic vesicles within a dense cluster can not be resolved if they are vertically stacked within the imaging volume. Therefore, with the approach described here, quantitative analyses of vesicle sizes and distributions are limited to volumes containing low densities of synaptic vesicles in the axial domain.

### Confocal - STED Microscopy Comparison of Synaptophysin Immunolabels in the Calyx Terminal

**Figure 4 pone-0062893-g004:**
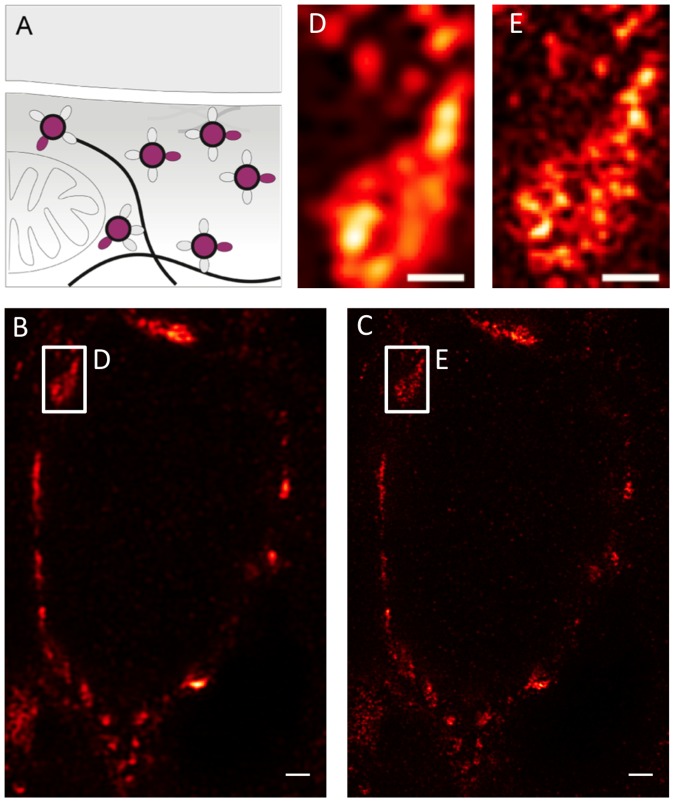
Nanoscopic synaptophysin distribution in the calyx of Held. (**A**) Schematic diagram of synaptophysin localization at synaptic release sites. (**B**) Confocal and (**C**) STED images of the synaptophysin distribution within an entire calyx cross section (see Figure 1 for illustration). Wiener filter applied to STED image. (**D,E**) Magnified views of the regions indicated in B,C. Scale bars 1 µm (**B,C**) and 500 nm with a pixel size of 19 nm (**D,E**)**.**

Having shown VGluT1 immunoreactivity at a resolution of single synaptic vesicles, we aimed at optimizing a staining protocol (see Methods) for subdiffraction imaging of the vesicle membrane glycoprotein synaptophysin (**Figure 4A**). We used a monoclonal antibody with specific reactivity against an epitope localized at the cytoplasmatic tail of isoform 1 (SYP1). A typical synaptic vesicle contains on average 32 synaptophysin molecules, constituting around 10% of the total vesicle protein, two times more than VGluT1 [8]. We visualized the distribution of SYP1 using confocal (**Figure 4B**) and STED (**Figure 4C**) microscopy. The STED image shows a clear increase in resolvable detail. This is further demonstrated in a region imaged at higher zoom (**Figure 4D,E**). In the confocal mode, neighboring vesicle pools can just about be resolved as separated structures, whereas STED microscopy reveals individual vesicles and a vesicular substructure within the cluster (**Figure 4D,E**).

### Nanoscopic Rab3A Distribution

**Figure 5:**
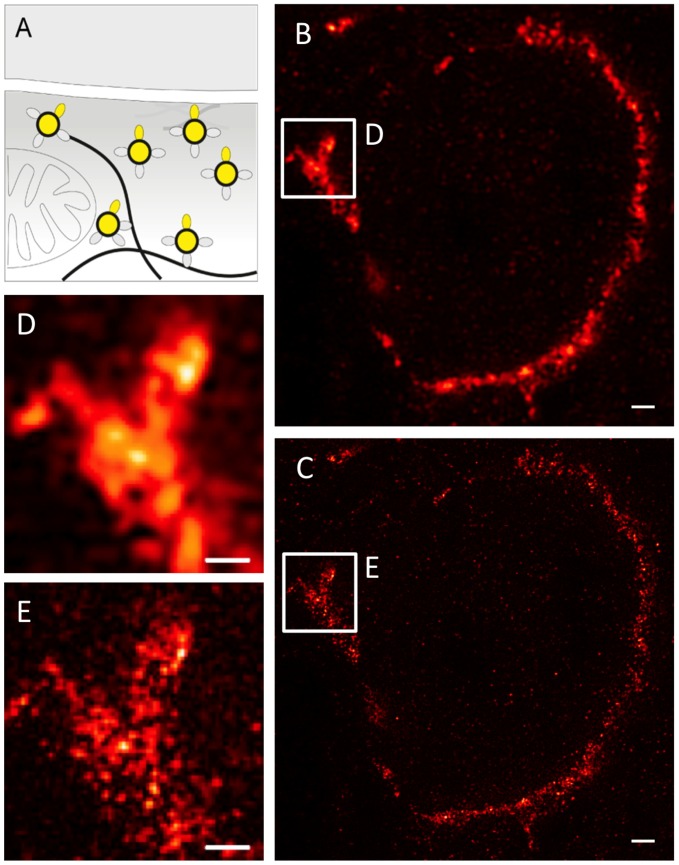
Nanoscopic Rab3A distribution in the calyx of Held. (**A**) Schematic diagram of the relative localization of Rab3A at synaptic release sites. (**B**) Confocal and (**C**) STED image of Rab3A distribution within a calyx cross-section. Wiener filter applied to STED image. (**D,E**) Magnified views of the regions indicated in B,C. Scale bars 1 µm. (**B,C**) and 500 nm with a pixel size of 19 nm (**D,E**).

We further investigated the distribution of Rab3A, which belongs to the ras-related superfamily of small monomeric GTPases. Small regulatory Rab GTPases are trafficking proteins that control membrane identity and regulate intracellular membrane transport in all eukaryotes. [9]. Rab3A is reversibly associated with the membrane of synaptic vesicles and is involved in the control of their targeting or docking at active zones (**Figure 5A**). It is highly abundant in the presynaptic terminal [10]. The confocal image of a Rab3A immunostain shows a characteristic calyceal staining pattern (see Figure 1) consisting of clusters occupying most of the calyceal volume (**Figure 5B,C**. Corresponding STED images show Rab3A immunosignals presumed to reflect individual synaptic vesicles or small clusters thereof (**Figure 5D,E**).

### Enhanced Resolution of STED Microscopy Reveals Heterogeneous Synapsin Distribution

**Figure 6:**
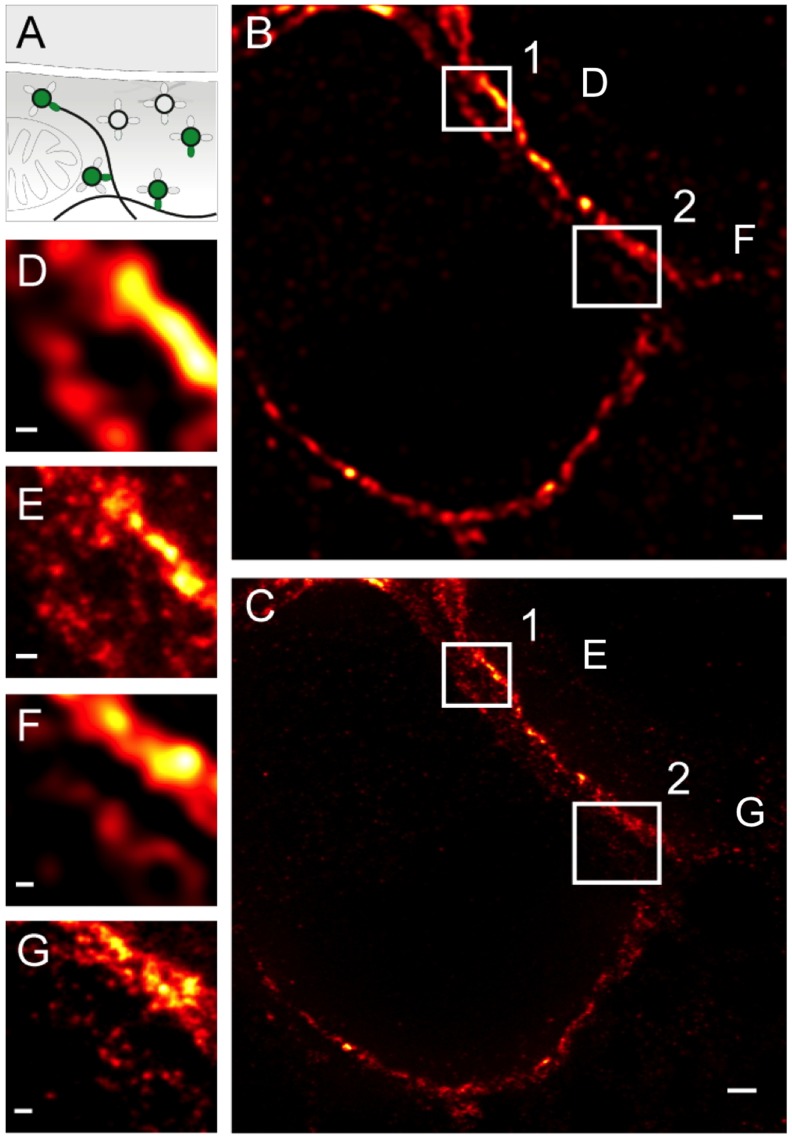
Nanoscopic synapsin distribution in the calyx of Held. (**A**) Schematic diagram of the presynaptic localization of synapsin. (**B,C**) Confocal and STED (**C,E,G**) images of synapsin immunosignal in presynaptic regions of the calyx of Held. Wiener filter applied to STED image (raw data for comparison in Supplemental Figure 1). (**D,E,F,G**) Magnified views of the regions indicated in B and C. Scale bars 1 µm in B,C and 200 nm in D–G with a pixel size of 19 nm.

Synapsins are vesicle-associated proteins involved in organizing a component of the reserve pool (**Figure 6A**) and in re-filling of the readily-releasable pool of synaptic vesicles [11], [12]. Confocal microscopy pointed towards distinct expression domains of synapsin (Vasileva and Kuner, unpublished observations), an observation that we further explored with STED microscopy. Except for some differences in fluorescence intensity, the confocal synapsin signal is rather uniformly distributed within three calyces partially positioned in a back-to-back configuration (**Figure 6B,D,F** **; for unfiltered data see Supplemental** **Figure 1**). However, STED microscopy revealed a clustered structure of the synapsin immuno-signal (**Figure 6C,E,G**) consistent with the vesiclular distribution pattern shown in **Figures 3** to [Fig pone-0062893-g004] 5. Hence, most of the synapsin immuno-signal appears to arise from synapsin molecules bound to synaptic vesicles, although synapsin may also exist in a freely floating cytoplasmic state not bound to synaptic vesicles.

### Dual-color Tissue STED Microscopy Reveals Synaptic Vesicles Lacking Synapsin

**Figure 7:**
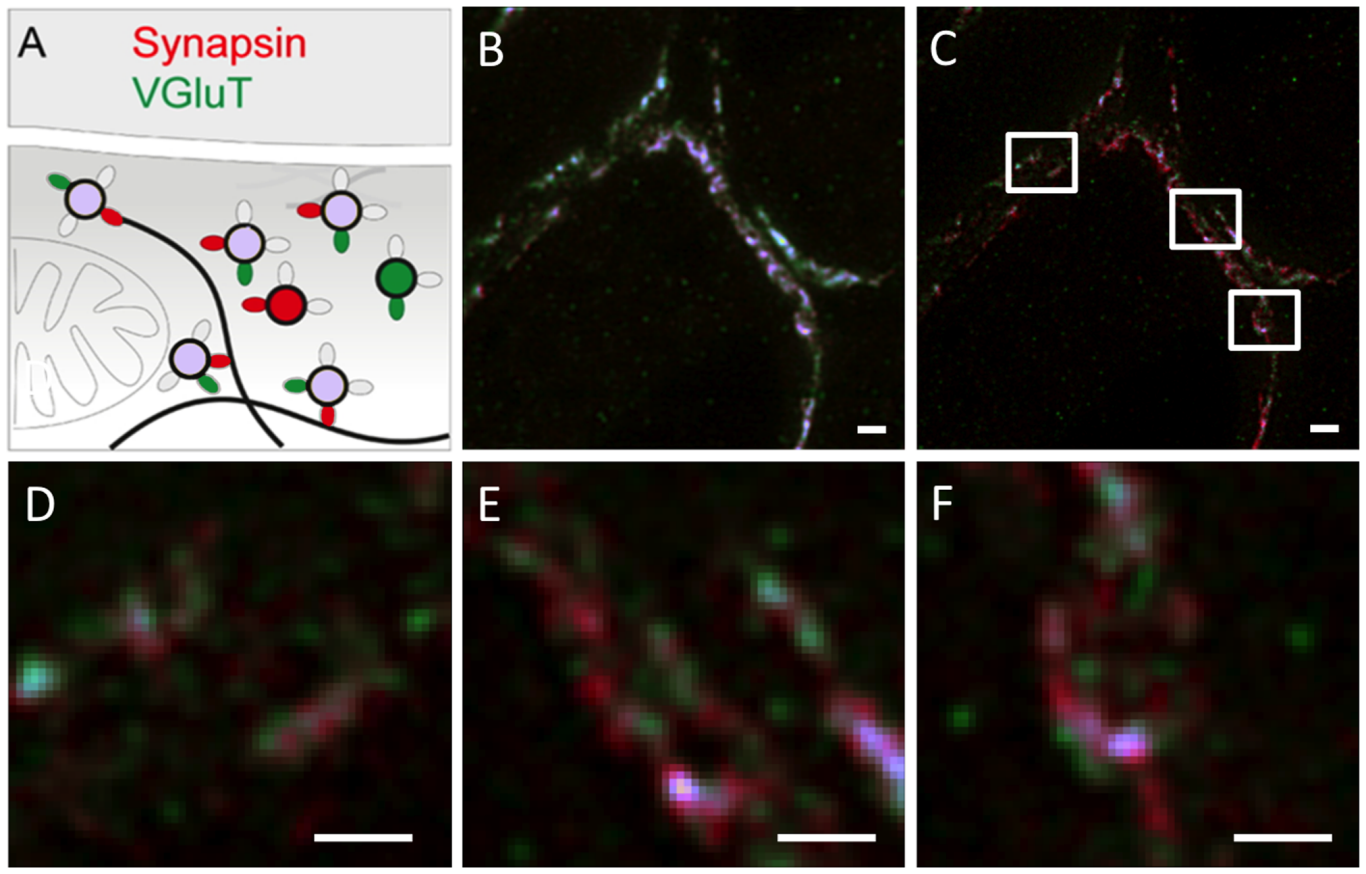
Dual color STED nanoscopy of synapsin and VGluT1 distribution in the calyx of Held. (**A**) Schematic diagram depicting the hypothesized interplay of synapsin and synaptic vesicles (VGluT1) in a glutamatergic terminal. (**B**) Dual-color confocal and (**C**) STED images of synapsin and VGluT1 distribution in the calyx. Scale bar 1 µm. (**D,E,F**) Magnified views of the subregions marked in **C** in clockwise order of their appearance. Scale bar 500 nm with a pixel size of 19 nm.

To further resolve the positioning of synapsin proteins relative to synaptic vesicles we stained slices against VGluT1 (Atto 565) and synapsin (Dyomics 485 XL) (**Figure 7A**). The confocal (**Figure 7B**) versus STED (**Figure 7C**) overview images show three neighboring calyces. While the confocal image suggests perfect colocalization of both proteins, the STED image reveals that synapsin (red) is only partially colocalized with VGluT1 (green) immuno-signal. Consistent with the data shown in Figure 6, synapsin immuno-labels show a mostly clustered distribution (**Figures 7D–F**). Notably, the larger clusters of synapsin signal colocalize with dense clusters of VGluT1-labeled vesicle signal (blue in Figures 7D–F). In addition, STED microscopy revealed nanodomains containing synaptic vesicles lacking synapsins (green). Conversely, some nanodomains contained synapsin but no VGluT1 reactivity (see Supplemental Figure 2 for images showing the individual channels). Despite the limited axial resolution (approximately 600 nm) of the imaging approach applied here, we conclude that non-overlapping expression domains exist, while conclusions on the co-localization of two fluorophores are limited to the STED microscopy focal volume of approximately 40 nm×40 nm×600 nm.

Hence, within presynaptic terminals a subset of synaptic vesicles exist that are not associated with synapsins. This differential tagging of synaptic vesicles may be a molecular correlate of synaptic vesicle pools [13]. In conclusion, we have proven the applicability of STED microscopy to reveal the spatial relationships of two functional proteins within a subdiffraction volume in aldehyde-fixed mammalian brain tissue.

## Discussion

### Experimental Conditions of STED Microscopy in PFA-fixed Mammalian Tissue

STED microscopy was applied to PFA-fixed mammalian brain slices. To obtain high-resolution STED signals in tissue, maintaining a perfect zero intensity distribution at the center of the doughnut-shaped beam within the tissue is of utmost importance. Tissue imaging is mainly restricted by light scattering, distorting the wave front and degrading signal and resolution. Absorption and scattering properties of multiple components are intrinsic properties of the tissue sample. In neural tissue, especially the brainstem, myelin causes strong light scattering. Hemoglobins and cytochromes residing after perfusion are responsible for most of the light absorption. Apart from the optical properties, homogenous antibody penetration, labeling specificity and signal density have to be considered, as well as pH and environmental influences on the physicochemical dye characteristics. We tackled these hurdles by optimizing immunostainings (see Materials and Methods) and by sectioning the tissue at a thickness of approximately 4 µm. The tissue was embedded in TDE [22] for fine adjustment of pH and refractive index. Furthermore, the antioxidant properties of TDA preserved the fluorescence quantum yield. In conclusion, this approach allowed us to image presynaptic proteins in the calyx of Held at a lateral resolution of 40 to 60 nm. Supported by the theoretical considerations shown in Figure 3, STED microscopy can discriminate single synaptic vesicles in PFA-fixed brain tissue.

### STED Microscopy Reveals Presynaptic Nano-architecture

We studied the distribution of several presynaptic integral synaptic vesicle proteins (VGluT, synaptophysin) and synaptic vesicle-associated proteins (Rab3A and synapsin). Synaptic vesicles contain multiple copies of each of these proteins at different stoichiometries (VGluT1 9, synaptophysin 32, synapsin 8, Rab3A 10 copies per vesicle [8]). Synapsin and Rab3A are abundantly associated with synaptic vesicles, but can also detach and exist cytosolically. Disconnection of synapsin is regulated by Ca^2+^-calmodulin-dependent phosphorylation [11], whereas Rab3A reversibly associates with vesicles in its GTP bound form [14], but detaches upon GTP hydrolysis during or after exocytosis [15]. At the level of confocal resolution, the immunosignals of the four proteins occupy most of the calyx volume and may form large clusters consistent with synaptic vesicles clusters [6], [12]. STED microscopy resolved the synaptic vesicle clusters at a much greater detail as fine punctiform patterns of locally varying density, as expected from electron micrographs of synaptic vesicles. Yet, STED microscopy of single two-dimensional frames did not suffice to find differences in distribution patterns between the integral synaptic vesicle proteins and vesicle-associated proteins examined here. As topographic relations between interacting proteins affect the dynamics of synaptic processes within the tissue, super-resolution measurements capable to reveal subcellular geometrical properties are required. Three-dimensionally resolved STED microscopy may provide a powerful approach to detect potential differences in distribution patterns of proteins on the nanoscale. Furthermore, also quantitative analyses of sizes and size distributions of fluorescence signals will require 3D STED imaging. This is due to the complexities of a 4 µm thick tissue section which causes the fluorophores to be distributed over a large volume. The target molecules will assume different positions relative to the focal plane, resulting in apparently different sizes. In conclusion, while 2D STED microscopy of brain tissue represents a significant advance over conventional fluorescence microscopy, an extention into the third dimension appears necessary to fully resolve the nano-architecture of subcellular compartments.

### Multiplexing at Nano-scale Resolution Allows Topological Mapping of Presynaptic Proteins

The spatial properties of synapses can span a wide range of morphological variability [18], thus the assessment of the precise spatial relationship of two or more synaptic components appears more important than a protein’s absolute distribution. We chose to probe the degree of overlap between synaptic vesicles labeled with anti-VGluT1 and anti-synapsin antibodies. The spatial correlation of the two selected signals at nanometer particle size should allow the detection of both, closely neighboring and partially colocalizing immunosignals (**Figure 7**). This could only be achieved by using fluorophores with an extra long Stokes-shifts (MegaStokes dyes), emphasizing that further advances in superresolution imaging of complex tissue are highly dependent on available fluorescent labels and sample preparation. The dye pair Atto 565 and Dyomics 485 XL represents a suitable combination that allows precise and stable excitation multiplexing at nanoscale resolution, allowing us to address the question if distinct synapsin-dependent and –independent synaptic vesicles and clusters thereof exist. We found three classes of immunosignals: isolated VGluT1, isolated synapsin and colocalized VGluT1/synapsin immunoreactivity (Figure 7). We interpret this as synaptic vesicles lacking synapsin, dispersed synapsin unbound to synaptic vesicles [16], [17] and synaptic vesicles with bound synapsin, respectively. The latter interpretation is constrained by the limited axial resolution of our imaging approach, providing the possibility that two or even more vesicles may be stacked vertically within the imaging volume. In this situation, one vesicle containing only synapsin and another one containing only VGluT1 would suggest colocalization of both proteins, although on the level of a single vesicle they would not colocalize. Nevertheless, vesicles devoid of vesicular glutamate transporters are unlikely to exist because the transporters are essential for presynaptic function and are expressed at a high abundance at the calyx of Held [19]. If either VGluT1 or synapsin can be detected in the volume sampled by STED microscopy, the conclusion seems warranted that either synaptic vesicles lacking synapsin or dispersed synapsin [16], [17] are present, respectively. However, in this case another limitation arsing from using antibodies for protein localization needs to be considered: steric hindrance and inaccessibiliy of the epitope may prevent binding of one of the antibodies. As a consequence, the absence of a signal may not necessarily reflect the absence of the protein. However, given that approximately 10 copies of each VGluT1 and synapsin exist per synaptic vesicle, it appears unlikely that these factors prevented detection of the respective protein. Therefore, we consider the findings reported here consistent with the existence of a synapsin-dependent and synapsin-independent component of the reserve pool [12], supporting the hypothesis that molecularly and structurally distinct synaptic vesicle pools operate in presynaptic nerve terminals. Hence, we demonstrated that the enhanced resolution provided by STED microscopy can reveal new synaptic protein topologies that have the potential to explain existing structural and functional properties of the synapse.

Our presented concept combines immunohistochemistry protocols optimized for labeling density and signal-to-noise-ratio with imaging schemes that allow for single- and multicolor tissue STED microscopy. Hereby, we are able to acquire information on the spatial relationships of different presynaptic proteins down to length-scales of ∼ 40 nm. Further optimizations of the axial resolution using serial sectioning approaches [20] or Iso-STED microscopy [21] promise insights into 3D protein topology with isotropic voxel sizes of 40 nm×40 nm×40 nm or better.

## Materials and Methods

### Animals and Tissue Preparation

All animal experiments were conducted in accordance with the German animal welfare guidelines. They were evaluated by the animal care and use committee of the state Baden-Württemberg and were approved by the state authorities (Regierungspräsidium Karlsruhe). Deeply anaesthetized Sprague-Dawley rats at postnatal ages P14-P16 were transcardially perfused with 40–60 ml isotonic 0.1 M phosphate buffered saline (PBS: 0.1 M Na_2_HPO_4_, 0.1 M NaH_2_PO_4_, pH 7.2) prior to fixation with 50 ml of 4% chilled paraformaldehyde. The brain was immediately removed from the skull and post-fixed for a period of 4 to 16 hours in the same fixative-solution at 4°C. Coronal tissue sections of 100 µm thickness where cut on a vibratome (HR2, Sigmann Elektronik). After indirect immunolabeling of the medial nucleus of the trapezoid body (MNTB) sections were infiltrated with 10% sucrose and 10% polyvinylpyrrolidone (PVP) in PBS for 4 hours, followed by 4 hours infiltration with 20% sucrose and 10% PVP in PBS, incubated for 12–16 hours in 30% sucrose with 10% PVP in PBS and finally frozen by submersion into liquid nitrogen. The trimmed tissue blocks extracted from the 100 µm sections were mounted on Tissue-Tek (Leica) for support and orientation and slowly cut on an ultramicrotome (Leica, Ultracut S; Cryochamber EM FCS) using a diamond knife (Histo Cryo 45°; Diatome, Schweiz). The slices had a thickness of approximately 4 µm. Sections were picked up with a perfect loop (Diatome) in 2.3 M sucrose in PBS and floated in PBS on ice. Prior to imaging, the slices were embedded in 2,2′-thiodiethanol (TDE). TDE is a nontoxic glycol derivative with antioxidant properties. The refractive index of a mixture of TDE and water can be continuously tuned from that of water (1.33) to that of immersion oil (1.52) [22].

### Optimizations of Tissue Sectioning

We tested the impact of tissue section thickness on the performance of STED microscopy imaging by testing sections with a thickness ranging from 50–100 µm to thin sections of 3 to 5 µm thickness. The 50–100 µm thick sections were produced with a vibratome while thin sections were made with a cryo-ultramicrotome using the thick sections as starting material. The thick sections were routinely tested for the quality of the immunostaining using confocal microscopy before generating thin sections from the same specimen. Typically, the superficial 10 to 20 µm of the slices were removed to discard regions with increased unspecific antibody binding. In summary, using this approach significantly improved imaging conditions for STED microscopy.

### Immunohistochemistry

After fixation, all labeling and further tissue processing was done rigidly at 4°C. Solutions and antibody dilutions were chilled to 4°C prior to application. Slices were incubated in blocking solution (BS): 0.5% t- octylphenoxypolyethoxyethanol (TX 100, Sigma-Aldrich), 5% bovine serum albumin (BSA; Sigma-Aldrich) and 2% teleostean gelatin (Sigma-Aldrich) in PBS for 90–120 min. Primary antibodies (all Synaptic Systems GmbH, Göttingen: anti-VGLUT 1 rabbit polyclonal, affinity purified (#135 303), dilution 1∶1500, anti-Synaptophysin 1 mouse monoclonal, purified IgG (#101 011), dilution 1∶2000, anti-Rab 3a mouse monoclonal, purified IgG (#107 111), dilution 1∶1000, Synapsin-I mouse monoclonal (#106 001), dilution 1∶1000, Bassoon rabbit polyclonal, affinity purified (#141 003), dilution 1∶1000) and both secondary antibodies labeled with Atto 565 (Atto-Tec, Siegen; dilution 1∶300) or Dyomics 485 XL (Dyomics GmbH, Jena; dilution 1∶100–1∶200) were diluted in 0.2% TX 100, 2% BSA and 2% teleostean gelatin in PBS and the slices were incubated for 12–14 hours. After primary antibody incubation, stringent rinsing (1×5 min, 2×10 min, 1–3×30 min) with PBS was applied. Secondary antibodies were also prepared in 0.2% TX 100, 2% BSA and 2% teleostean gelatin in PBS. Sections were incubated with secondary antibody for 12–14 hours after a 90–120 min incubation in blocking solution (BS). All incubation was done in 24-well-plates in a total volume of 500 µl at 250 rpm on a vibrating platform shaker (Vibramax 100, Heidolph-Instruments, Schwabach). Sections were rinsed in PBS for at least 3 hours before infiltration with 10% sucrose for further tissue processing.

### Optimizations of Immunohistochemical Procedures

General procedural optimizations of immunohistochemistry included the fresh preparation of solutions for each set of experiments, prechilling of the solutions to 4°C prior to use, keeping the tissue samples always at 4°C, blocking for 2 h using a combination of teleostean gelatin and BSA, continuous agitation of the sections in all washing, blocking and incubation steps, increased number of washing steps (up to 5) and determination of ideal antibody dilutions by serial dilution and subsequent analysis using confocal microscopy (typically dilutions ranging from 1∶100 to 1∶10000 were tested).

### Dyes

To optimize fine-structure detection we used dyes resistant to photobleaching with large extinction coeffients and quantum yields. For single-color imaging, we used the rhodamine derivate Atto 565 (*Abs: 563* *nm, Em: 592* *nm å: 120,000 M^−1^cm^−1^, QY 90*). In the experimental setting for dual-color STED microscopy, we additionally chose Dyomics 485 XL (*Abs: 485 nm, Em: 560 nm, absorbtion coefficient: 50,000 M^−1^ cm^−1^)* displaying an exceptionally large wavelength shift (Stoke's shift) between its excitation and emission maximum. This allowed the usage of two separate excitation laser beams and one common depletion laser source for both fluorophores for STED nanoscopy.

### Imaging and Image Processing

Pulsed excitation of the Atto565 (Atto-Tec, Siegen, Germany) labeled antibodies was achieved using a high-repetition rate laser diode source (PicoTA, Picoquant, Berlin, Germany) at a wavelength of λ_exc_ = 532 nm (pulse length <100 ps, spectral width <1 nm), synchronized with the STED-laser via a fast photodiode (Alphalas, Göttingen, Germany). Dy-485XL (Dyomics GmbH, Jena, Germany) was excited by a λ_exc_ = 470 nm pulsed high-repetition rate laser diode source with a power-dependent pulse length of 90–300 ps and spectral width of 1–2 nm (Picoquant, Berlin, Germany). An actively mode-locked (APE, Berlin, Germany) Ar-Kr laser (Spectra Physics-Division of Newport Corporation, Irvine, USA) was used as STED laser at λ_STED_ = 647 nm (pulse length 200–300 ps, spectral width <<1 nm). All beams were combined using acousto-optical tunable filters (AOTF) (Crystal Technologies, Palo Alto, USA) and coupled into a microscope stand (DMI 4000B, Leica Microsystems GmbH, Mannheim, Germany) equipped with a three axis piezo stage-scanner (PI, Karlsruhe, Germany). The AOTFs enabled blanking of the lasers and allowed the power of each laser beam to be controlled independently. This allowed the blanking of the scanner fly back via the line signal from the data acquisition software (Imspector, MPI Göttingen, Germany). They also provided a means for selecting counter-propagating fluorescence returning from the microscope. The collected fluorescence passed through an additional band-pass filter (580/40 for Atto565 and Dy-485XL, AHF Analysentechnik, Tübingen, Germany) and was detected confocally with a photon counting module (SPCM-AQR-13-FC, PerkinElmer, Canada). A vortex phase plate (RPC Photonics, NY, USA) was used in the STED beam path to generate a doughnut-shaped beam in the focus. To determine the resolution of the microscope, a sheep anti-mouse IgG antibody labeled with Atto565 was sparsely adsorbed to a glass surface and embedded in Mowiol yielding single point-like objects. A single-step linear deconvolution (LD), i.e. Wiener filter, was carried out with a theoretical PSF of 40 nm FWHM for STED images and 200 nm for confocal images. A comparison of the linear deconvoluted data to the unprocessed raw scans can be appreciated in **Figure S1.** We ran several experimental staining series, defining the best blocking combinations to minimize unspecific low-affinity binding to the tissue and to determine the least possible antibody concentrations. To avoid sampling of nonrepresentative and insufficiently labeled MNTB sections, we confocally imaged whole stacks of calyces to confirm complete and homogeneous labeling within the entire calyx, regardless of penetration depth, for each antibody staining protocol.

## Supporting Information

Figure S1 **Raw data of calyx overview scans corresponding to** **Figure 6** **.** (**A**) Schematic diagram of the presynaptic localization of synapsin. (**B**) Unprocessed confocal and (**C**) STED scan of synapsin signal in the calyx. (**D,E,F,G**) Magnified views of the regions indicated in B and C. Scale bars 1 µm in B,C and 200 nm in D–G.
(TIF)

Figure S2 **Dual color STED images corresponding to** **Figure 7** **shown as individual channels.** The RGB panels are taken from Figure 7. The individual channels red (R = synapsin), green (G = VlguT) and blue (B = overlap) illustrate that many signals in the green channel do not have a correspondence in the red channel. Wiener filter applied in all panels. Scale bars 500 nm.
(TIF)

## References

[1] Kittel RJ, Wichmann C, Rasse TM, Fouquet W, Schmidt M, Schmid A, et al. (2006). Bruchpilot promotes active zone assembly, Ca^2+^ channel clustering, and vesicle release. Science 312, 1051–1054. 10.1126/science.1126308 16614170

[2] Liu KSY, Siebert M, Mertel S, Knoche E, Wegener S, Wichmann C et al.. (2011). RIM-binding protein, a central part of the active zone, is essential for neurotransmitter release. Science, 334, 1565–1569. 10.1126/science.1212991 22174254

[3] Südhof TC (2012) The Presynaptic Active Zone. Neuron 75: 11–25. 22794257 10.1016/j.neuron.2012.06.012 PMC3743085

[4] Hell SW, Wichmann J (1994) Breaking the diffraction resolution limit by stimulated emission: stimulated-emission-depletion fluorescence microscopy. Opt Lett 19: 780–782. 19844443 10.1364/ol.19.000780

[5] Sätzler K, Söhl LF, Bollmann JH, Borst JGG, Frotscher M, et al (2002) Three-Dimensional Reconstruction of a Calyx of Held and Its Postsynaptic Principal Neuron in the Medial Nucleus of the Trapezoid Body. The Journal of Neuroscience 22: 10567–10579. 12486149 10.1523/JNEUROSCI.22-24-10567.2002 PMC6758464

[6] Dondzillo A, Sätzler K, Horstmann H, Altrock WD, Gundelfinger ED, et al (2010) Targeted three-dimensional immunohistochemistry reveals localization of presynaptic proteins Bassoon and Piccolo in the rat calyx of Held before and after the onset of hearing. The Journal of Comparative Neurology 518: 1008–1029. 20127803 10.1002/cne.22260

[7] Borst JGG, Soria van Hoeve J (2012) The Calyx of Held Synapse: From Model Synapse to Auditory Relay. Annual Review of Physiology 74: 199–224. 10.1146/annurev-physiol-020911-153236 22035348

[8] Takamori S, Holt M, Stenius K, Lemke EA, Grønborg M, et al (2006) Molecular Anatomy of a Trafficking Organelle. Cell 127: 831–846. 17110340 10.1016/j.cell.2006.10.030

[9] Stenmark H (2009) Rab GTPases as coordinators of vesicle traffic. Nat Rev Mol Cell Biol 10: 513–525. 19603039 10.1038/nrm2728

[10] Pavlos NJ, Jahn R (2011) Distinct yet overlapping roles of Rab GTPases on synaptic vesicles. Small GTPases 2: 77–81. 21776405 10.4161/sgtp.2.2.15201 PMC3136907

[11] Cesca F, Baldelli P, Valtorta F, Benfenati F (2010) The synapsins: Key actors of synapse function and plasticity. Progress in Neurobiology 91: 313–348. 20438797 10.1016/j.pneurobio.2010.04.006

[12] Vasileva M, Horstmann H, Geumann C, Gitler D, Kuner T (2012) Synapsin-dependent reserve pool of synaptic vesicles supports replenishment of the readily releasable pool under intense synaptic transmission. European Journal of Neuroscience: no-no. 10.1111/j.1460-9568.2012.08225.x 22805168

[13] Denker A, Rizzoli SO (2010) Synaptic vesicle pools: an update. Frontiers in Synaptic Neuroscience 2. 10.3389/fnsyn.2010.00135 PMC3059705 21423521

[14] Mizoguchi A, Kim S, Ueda T, Kikuchi A, Yorifuji H, et al (1990) Localization and subcellular distribution of smg p25A, a ras p21-like GTP-binding protein, in rat brain. Journal of Biological Chemistry 265: 11872–11879. 2114404

[15] von Mollard GF, Sudhof TC, Jahn R (1991) A small GTP-binding protein dissociates from synaptic vesicles during exocytosis. Nature 349: 79–81. 1845915 10.1038/349079a0

[16] Orenbuch A, Shulman Y, Lipstein N, Bechar A, Lavy Y, et al (2012) Inhibition of exocytosis or endocytosis blocks activity-dependent redistribution of synapsin. Journal of Neurochemistry 120: 248–258. 22066784 10.1111/j.1471-4159.2011.07579.x

[17] Denker A, Kröhnert K, Bückers J, Neher E, Rizzoli SO (2011) The reserve pool of synaptic vesicles acts as a buffer for proteins involved in synaptic vesicle recycling. Proceedings of the National Academy of Sciences 108: 17183–17188. 10.1073/pnas.1112690108 PMC3193190 21903923

[18] Welzel O, Henkel AW, Stroebel AM, Jung J, Tischbirek CH, et al (2011) Systematic Heterogeneity of Fractional Vesicle Pool Sizes and Release Rates of Hippocampal Synapses. Biophysical Journal 100: 593–601. 21281573 10.1016/j.bpj.2010.12.3706 PMC3030169

[19] Punge A, Rizzoli SO, Jahn R, Wildanger JD, Meyer L, et al (2008) 3D reconstruction of high-resolution STED microscope images. Microscopy Research and Technique 71: 644–650. 18512740 10.1002/jemt.20602

[20] Billups B (2005) Colocalization of vesicular glutamate transporters in the rat superior olivary complex. Neuroscience letters 382: 66–70. 15911123 10.1016/j.neulet.2005.02.071

[21] Schmidt R, Wurm CA, Jakobs S, Engelhardt J, Egner A, et al (2008) Spherical nanosized focal spot unravels the interior of cells. Nat Meth 5: 539–544. 10.1038/nmeth.1214 18488034

[22] Staudt T, Lang MC, Medda R, Engelhardt J, Hell SW (2007) 2,2′-Thiodiethanol: A new water soluble mounting medium for high resolution optical microscopy. Microscopy Research and Technique 70: 1–9. 17131355 10.1002/jemt.20396

