# Supplementary figures and images for: Tissue Multicolor STED Nanoscopy of Presynaptic Proteins in the Calyx of Held
Source: PLoS One. 2013 Apr 26;8(4):e62893. doi: 10.1371/journal.pone.0062893 (PMC3637247; doi:10.1371/journal.pone.0062893)

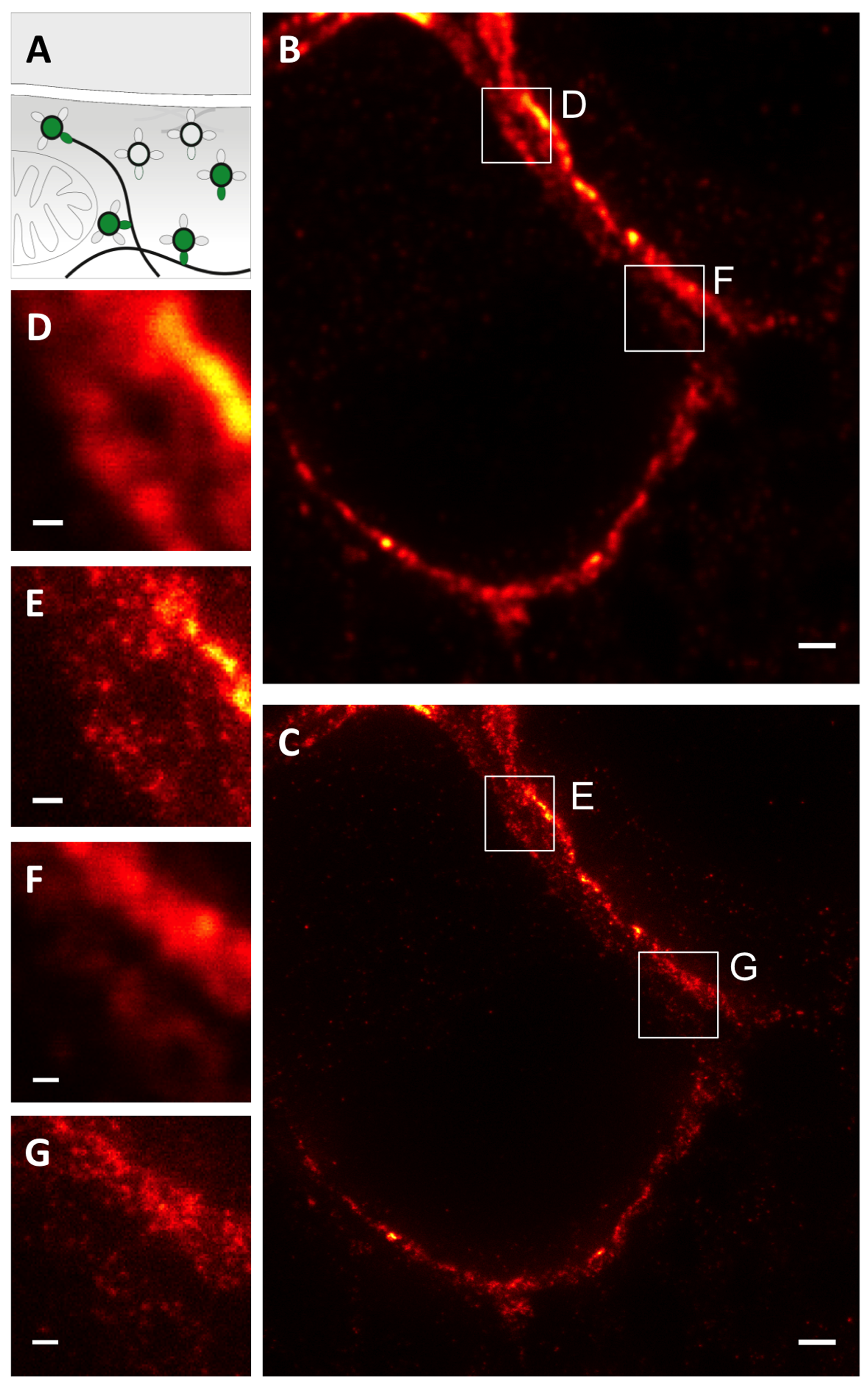

Supplement: Figure S1 — Raw data of calyx overview scans corresponding to Figure 6 . (A) Schematic diagram of the presynaptic localization of synapsin. (B) Unprocessed confocal and (C) STED scan of synapsin signal in the calyx. (D,E,F,G) Magnified views of the regions indicated in B and C. Scale bars 1 µm in B,C and 200 nm in D–G. (TIF) [file pone.0062893.s001.tif]

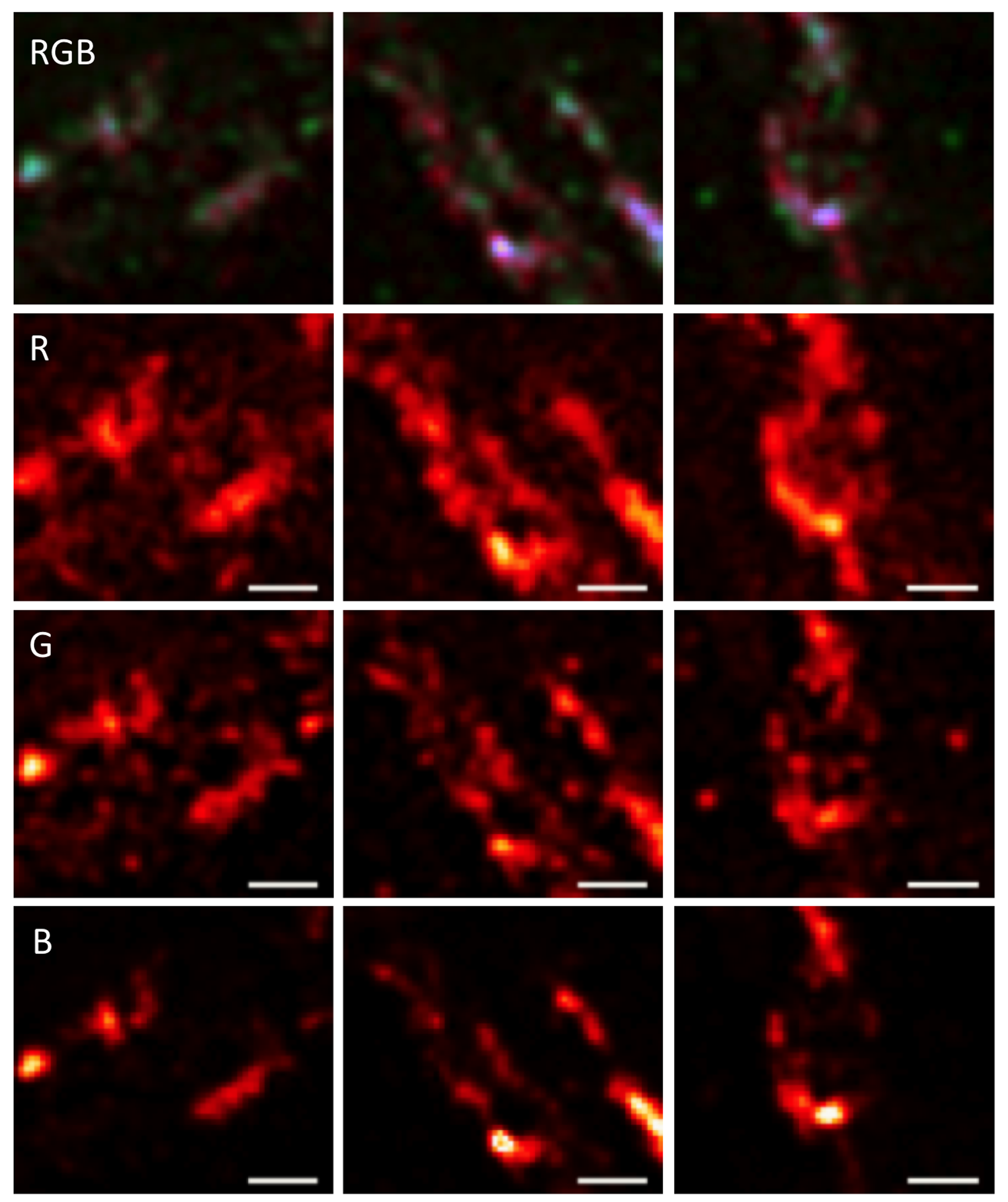

Supplement: Figure S2 — Dual color STED images corresponding to Figure 7 shown as individual channels. The RGB panels are taken from Figure 7. The individual channels red (R = synapsin), green (G = VlguT) and blue (B = overlap) illustrate that many signals in the green channel do not have a correspondence in the red channel. Wiener filter applied in all panels. Scale bars 500 nm. (TIF) [file pone.0062893.s002.tif]
